# Letter to the editor: *Chlamydia trachomatis* samples testing falsely negative in the Aptima Combo 2 test in Finland, 2019

**DOI:** 10.2807/1560-7917.ES.2019.24.24.1900354

**Published:** 2019-06-13

**Authors:** Magnus Unemo, Damon Getman, Ronza Hadad, Michelle Cole, Nicholas Thomson, Mirja Puolakkainen, Gianfranco Spiteri

**Affiliations:** 1 WHO Collaborating Centre for Gonorrhoea and other STIs, National Reference Laboratory for STIs, Department of Laboratory Medicine, Faculty of Medicine and Health, Örebro University, Örebro, Sweden; 2 Hologic Inc., San Diego, CA, United States; 3 National Infection Service, Public Health England, London, United Kingdom; 4 Wellcome Sanger Institute, Wellcome Genome Campus, Hinxton, United Kingdom; 5 Department of Virology and Immunology, University of Helsinki and Helsinki University Hospital, Huslab, Helsinki, Finland; 6 European Centre for Disease Prevention and Control (ECDC), Stockholm, Sweden

**Keywords:** *Chlamydia trachomatis*, Aptima Combo 2, Hologic, Finland, Nucleic Acid Amplification Test (NAAT)

**To the editor:** We read with great interest the recent article by Rantakokko-Jalava et al. about false-negative *Chlamydia trachomatis* (CT) specimens in the nucleic acid amplification test (NAAT) Aptima Combo 2 assay (AC2; Hologic Inc.) [1]. These very important findings indicate that 6–10% of CT-positive cases in Finland might have been missed due to false-negative/equivocal AC2 results. The AC2 false-negative/equivocal CT specimens were observed in several Finnish settings, particularly in south-western Finland, June 2018–April 2019. Finnish experts, together with Hologic scientists, quickly determined that the specimens with equivocal/negative CT results in AC2 (target: 23S rRNA) but positive in the Aptima *Chlamydia trachomatis* assay (ACT; target: 16S rRNA) were not linked to Hologic instruments or reagents. Concurrently, Finnish scientists identified a C1515T mutation in the CT 23S rRNA gene in 10 AC2 false-negative specimens [1]. Further investigation and response is required and will need collaborative action.

Hologic has now provided strong evidence that the C1515T mutation is the root cause of the AC2 false-negative/equivocal CT specimens and confirmed in analytical studies that a synthetic RNA transcript corresponding to the CT 23S rRNA with a C1515T mutation yields significantly suppressed CT detection probe-signal in AC2 similar to that observed with AC2 testing of clinical samples containing the mutated CT strain.

It is essential that a broader understanding is developed of the presence and proportion of specimens with mutated CT in Finland and internationally. Sufficient numbers of representative, geographically diverse false-negative AC2 specimens need to be examined in order to exclude the unlikely possibility of additional 23S rRNA mutations affecting detection. Using ACT for confirmation of AC2 screened specimens provides clinical utility in the short term but is not a feasible long-term solution, because of the high cost and substantially increased work load and time for reporting results imposed by confirming a large number of AC2 equivocal/negative specimens by ACT testing. Additionally, the different performance characteristics of AC2 and ACT may further confound our understanding of the presence, spread and possible evolution of the new mutant (or, although unlikely, mutants). This might even result in the use of only ACT in some settings, which would create problems due to the widespread use of AC2 as well as loss of information regarding the presence of mutated specimens, presence of *Neisseria gonorrhoeae*, and the advantage of reserving ACT for confirmation testing.

Instead, until a revised version of the AC2 test is available, a validated and quality-assured ‘mutant-specific’ research-use-only test is urgently needed to understand the geographic distribution of the CT mutant and its prevalence in screening populations. To fulfil this need, Hologic is rapidly developing an Aptima-format research kit containing a 23S rRNA-mutant detection probe. Surveillance using a test that detects the CT mutant will provide further timely insight into the impact of this CT variant on the effectiveness of CT screening in Finland and additional European countries where elevated levels of AC2 false-negative/equivocal results might be observed. In addition, this mutant-specific assay will generate evidence-based modifications to the current AC2 assay to ensure accuracy of test results and patient safety. The European Centre for Disease Prevention and Control (ECDC) intends to facilitate such preferably centralised study or studies which will require close collaboration with Finland, additional European Union/European Economic Area countries, other international and national public health organisations, and Hologic.

The key public health priorities include understanding the spread of the CT mutant and ensuring that patients with false-negative results are recalled and tested using alternative NAAT. To address the former, any unexplained changes in CT epidemiology and positivity rate need to be further investigated. In laboratories using AC2, retrospective and prospective review of the AC2 test results (proportions of positive, equivocal, negative test results, and associated relative light units (RLU) of test results) and investigations of any unexplained changes in the CT positivity rate should be performed. In settings with suspected false-negative CT test results by AC2, to ensure patient safety, specimens with low CT RLU values (≥15–99), independent of the result interpretation in the Hologic instruments, need retesting with an appropriate NAAT targeting another genetic sequence (preferably ACT), and not by repeated AC2 testing. The look-back period for recalling patients with verified false-negative results will depend on the local proportion and epidemiology of the CT mutant; taking into account spontaneous clearance of CT infection, social consequences for the patients, potential risk of reinfection, and continuous analysis of positivity rates of retested patients.

Investigation of additional scientific issues will be important in due course, e.g. if it is only one mutated CT clone; the emergence (when, where and how), transmission and evolution of the clone(s) in different populations in Finland and possibly other countries; if the clone(s) has(ve) any fitness advantage/disadvantage; if only one or both the CT 23S rRNA gene alleles are mutated in all specimens and how this affects the number or proportion of mutated 23S rRNA molecules detected in AC2; if the lack of AC2 detection of the clone(s) has increased risk of CT-associated complications/sequelae; associations between the clone(s) and symptomatic/asymptomatic infection, and spread in different subpopulations (patient sex, age, sexual orientation, geographic distribution etc.). Whole genome sequencing from false-negative AC2 CT specimens is in progress to address several of these issues. Furthermore, a mutant-specific real-time PCR is also under development.

Finally, it is likely that mutants of CT and other infectious agents escaping detection in commercial and laboratory-developed diagnostic NAATs are more common than previously realised and are inevitable consequences of the microbial evolution combined with the high diagnostic selective pressure on the microorganisms. Several lessons on such diagnostically-selected evolution can be learnt from the identification of the Swedish new variant of CT (nvCT) in 2006 and its subsequent spread mainly in Sweden [2-5]. International and national surveillance programmes capturing diagnostic test escape mutants (as well as cross-reacting mutants of other pathogens) for CT and other pathogens should be considered, particularly for diagnostic NAATs with a single genetic target region.
